# Interaction Patterns of Nurturant Support Exchanged in Online Health Social Networking

**DOI:** 10.2196/jmir.1824

**Published:** 2012-05-03

**Authors:** Katherine Y Chuang, Christopher C Yang

**Affiliations:** ^1^College of Information Science and TechnologyDrexel UniversityPhiladelphia, PAUnited States

**Keywords:** Social support, social media, alcoholism

## Abstract

**Background:**

Expressing emotion in online support communities is an important aspect of enabling e-patients to connect with each other and expand their social resources. Indirectly it increases the amount of support for coping with health issues. Exploring the supportive interaction patterns in online health social networking would help us better understand how technology features impacts user behavior in this context.

**Objective:**

To build on previous research that identified different types of social support in online support communities by delving into patterns of supportive behavior across multiple computer-mediated communication formats. Each format combines different architectural elements, affecting the resulting social spaces. Our research question compared communication across different formats of text-based computer-mediated communication provided on the MedHelp.org health social networking environment.

**Methods:**

We identified messages with nurturant support (emotional, esteem, and network) across three different computer-mediated communication formats (forums, journals, and notes) of an online support community for alcoholism using content analysis. Our sample consisted of 493 forum messages, 423 journal messages, and 1180 notes.

**Results:**

Nurturant support types occurred frequently among messages offering support (forum comments: 276/412 messages, 67.0%; journal posts: 65/88 messages, 74%; journal comments: 275/335 messages, 82.1%; and notes: 1002/1180 messages, 84.92%), but less often among messages requesting support. Of all the nurturing supports, emotional (ie, encouragement) appeared most frequently, with network and esteem support appearing in patterns of varying combinations. Members of the Alcoholism Community appeared to adapt some traditional face-to-face forms of support to their needs in becoming sober, such as provision of encouragement, understanding, and empathy to one another.

**Conclusions:**

The computer-mediated communication format may have the greatest influence on the supportive interactions because of characteristics such as audience reach and access. Other factors include perception of community versus personal space or purpose of communication. These results lead to a need for further research.

## Introduction

The Internet is a tool that can quickly connect people to each other, forming niche communities that house conversations. Many people go online for communicative or social reasons, as well as tailored information, as they face important decisions [1-4]. In 2009, the Pew Internet & American Life Project found that e-patients wanted to access user-generated or “just-in-time someone-like-me” health information such as newsgroups, blogs, social networking sites, or updates [4]. It is very likely that they are looking for compassion or experiential knowledge.

E-patients are Internet users who seek, share, and sometimes create information about health and wellness [4]. They benefit from sharing their experiences, discussing medical information, and exchanging social support. Social support through peer communication can enable e-patients to cope with stress, and it also increase access to information [5,6]. Emotional support, which is one type of social support, can lead to improved health outcomes such as easing adjustment to cancer [7]. Many users join online support groups for a sense of community around those who have similar experiences [7,8]. In fact, some researchers suggest that online communities become surrogate families of e-patients, where members share common problems and help each other toward mutual goals through good times and bad [7,8].

Online communities exist across websites built upon social technologies, such as bulletin boards and mailing lists, and social network sites. Social network sites are different from previous text-based communication formats in their emphasis on the website representing relationships between users and architectural elements that encourage interpersonal relationships [9]. Social network sites enable users to find each other and build connections using profile pages and a spectrum of private and public communication tools [10,11]. These social technologies are particularly useful for health services in enabling the creation of Web interventions to help heavy drinkers [12-14].

Studying the patterns of nurturing interactions within a support community gives useful insight into users’ information and emotional needs from an online support group. Each computer-mediated communication format has a unique combination of characteristics that affect the interaction patterns of nurturant support. Understanding the relation between the technology and how it affects user behavior could help both end users and designers of these online communities. Supposing that one computer-mediated communication format may have a higher association with a particular type of question, this could be useful in the design of Web interventions that incorporate online communities [15]. Studies such as the present one that report social interaction patterns in an online community could reveal areas of improvement for software that serves as the backbone of online communities. The objective of our research was to identify the types of nurturing social support in an online health care social networking site across three different text-based communication tools: discussion forums, personal journals, and notes. This study presents empirical data as evidence that patterns of nurturing support vary among the different online tools. We discuss our results in conjunction with theories to explain possible causes of support behavior patterns.

### Related Work

Previous research documented examples where e-patients needed online social support beyond technical support and information from health providers (eg, doctor visit reminders); however, these studies were limited on the technical front due to overlooking specific characteristics in the computer-mediated communication format as a factor of support group behavior. Computer-mediated communication enables interpersonal communication in a public environment, but end users often conduct their conversations on this platform as a private space [9,16-18]. This dilemma presents the motivation to compare supportive communication across multiple formats.

### Online Social Support

Support communities are likely to exist in a health domain when patients experience stress or confusion at a personal level [19]. E-patients’ information needs often coexist with other conditions (eg, receiving unfortunate news or requiring a behavior change) For example, a patient with liver cancer may have learned that the cancer was a result of overdrinking over many years. When shifting to a sober lifestyle, the patient may find general social support from friends to be helpful in preventing a relapse [20].

Of the different social support types available (informational, nurturant, and instrumental), nurturant support is known as a more intimate type of support, built upon trust and more frequent interactions [21,22]. It includes expressions of caring for someone without necessarily seeing tangible efforts. Social network sites are online spaces where users can work on creating intimate relationships with each other using the friending and messaging features, thus exchanging social support [10,23]. Expressing emotion is an important component in the daily management of relationships. Unlike in other online communities, users of social network sites tend to expect to gratify their social–emotional needs rather than informational needs [22]—for instance, positive comments between friends on general social network sites such as MySpace [23]. Close friends are sources of emotional support that can help with coping in difficult times or to improve mental health [24-26]. People with strong ties often communicate through many channels [24,27], but also with more emotional content than when communicating with strangers. We suspect that the different communication formats can reveal a spectrum of nurturant support types.

Positive outcomes from participation in self-help groups include (1) sharing information such as ideas, facts, and resources, (2) engaging in dialogue to reveal multiple perspectives, (3) discussing taboo subjects, (4) being “all in the same boat” with others, (5) experiencing mutual support, (6) engaging in problem solving and rehearsing, (7) overcoming alienation and isolation, (8) engaging in catharsis, (9) taking on the role of helper, (10) developing inspiration and hope, (11) developing social networks, and (12) assisting more people less expensively [8,19,28,29]. Support community members often share personal experiences, which may also include personal information. Emotional support is a valuable element of social support and in helping support seekers in coping with health problems [19,28,29]. Peer communication such as establishing social norms or finding role models and sharing feelings can also play a role in facilitating new health habits, such as quitting smoking [30]. Because of the nature and helpfulness of emotional support, we explored the nurturing types (esteem, network, and emotional) across the various computer-mediated communication formats.

### Medical Environment of Sharing Nurturant Support

While similar studies over the past decade also identified the content within online support communities across several health conditions, they did not focus on the impact of technology on the communication. Table 1 [6,19,21,28, 31-36] shows a brief list of these related studies. For instance, they compared types of social support exchanged across email lists and discussion groups [19,21,28,31] and bulletin boards [6,32-36]. These earlier forms of social technologies lacked some features of current social media computer-mediated communication formats, particularly profile pages.

**Table 1:** Previous studies of online support in health-related social networking sites.

Study	Data set
McCormack [6]	Eating disorder
Preece [19]	Torn knee ligament, 500 messages, April 1996–April 1997
Bambina [21]	Support OnLine Cancer Forum, 84 members,1149 messages
Braithwaite et al [28]	Support Network for disabilities, 42 users, 1472 messages
Meier et al [31]	Association of Cancer Online Resources
Cunningham et al [32]	Alcoholism, 10 months, 474 posts (moderated)
Coursaris et al [33]	HIV/AIDS^a^, 5000 messages
Eichhorn [34]	5 eating disorder message boards, 490 messages
Pfeil and Zaphiris [35]	Depression/seniors, discussion forum
Selby et al [36]	StopSmokingCenter.net (moderated) (November 6, 2004–May 15, 2007

^a ^Human immunodeficiency virus/acquired immunodeficiency syndrome.

Previous studies of online support communities also identified various types of support that they provided [6,19,21,28,32-35], and compared online support with face-to-face empathy [35] and with other types of communities [19]. They gave examples of benefits from participation [8,37]; however, these studies did not discuss their findings in light of communication format characteristics or looking at the relationship between the use of the tool and the tool itself. We were interested in a more in-depth study of social software features that affect social interaction patterns.

Some of these studies focused on evaluating support communities [32,36], while many of the other studies aimed to describe the content of the support communities [19,21,28,35]. Some studies also investigated the types of social support exchanged using Cutrona and Suhr’s [38] social support framework [33,34]. Evaluation could be useful in understanding what users need from the online support community.

### Computer-Mediated Communication Through Social Network Sites

We viewed social support exchanges in an online community from an architectural perspective, where the site design affects user behavior [9,39,40]. Certain features, such as privacy level settings, suggest acceptable behaviors to promote “the development of particular culture or behaviors and identity presentation;” however, users will customize them to improve their social interactions [9,39]. This might be a cause for concern because electronic media lack clear boundaries between traditionally public and private spaces [9,40]. In the physical world, the walls of offices and houses clearly separate distinct situations, and gates section off personal property. However, the Internet blurs the separation between public and private information in the online space [9]. In social network sites, users must balance their private and public selves, especially when conducting social interactions with each other. For example, Facebook is similar to a greenhouse, with its publicly open structure and many communication tools that members use to leave social cues for each other [9]. Public comments and other communication can signal the “strength and context of a relationship” [39].

Social network sites encourages disparate individuals to connect, communicate, and take action, which fosters interaction that is primarily interpersonal [9,41]. Social network sites provide the capability for users to represent themselves with an online presence (identity information) that contains shareable personal information, such as their birthday, preferences, photographs, and writings, and can assist in developing common ground and facilitate initial interactions [11,41]. Convenient features allow users to form and maintain online network “friends,” where, if one user invites another user with a friend request and is accepted, a relationship is established on the website [10]. Friends can communicate through social network sites in several ways, including private and public messaging systems [9,10,23,26,41]. Studying these interactions (eg, the length, frequency, and content of these comments) rather than explicit articulations on profile pages can reveal the conversational profile of each relationship [9,39].

Social network site interactions are founded upon norms of everyday face-to-face interaction, but when they are adapted to the online setting, the distinction between public and private spheres is blurred [17]. The space experience comes from relations with others [42,43]. In neutral spaces such as urban spaces that offer public gatherings, a group’s main activity is informal conversation. Public spaces such as parks are communal and have certain purposes of use compared with a private space such as a home. Communication tools are designed with a spectrum of privacy options, where each computer-mediated communication format may project toward users a different perspective of intimacy, and therefore lead to different social supports being offered and sought. For instance, users might post questions with factual answers in a forum that all other users can easily notice and access, for a better chance to receive a quick answer from a broad audience. On the other hand, users who seek encouragement may prefer using a personal journal to limit support requests to a smaller group of closely related users.

The research literature also suggests that, regardless of context, software features affect user behavior in online communities [9,16]. More specifically, elements within each computer-mediated communication format affect the resulting social interactions. In this study, we observed interactions in the forum, across user journals, and in profile posts on MedHelp. The forum is similar to a community hall in the sense that the space belongs to the community. In that community space, anyone can post in, comment on, or read discussion threads. All forum post and comments of a medical support community are available in the corresponding support community page. The threads are sorted by the dates when they are posted. The access authorization is managed by the community administration. In contrast to the forum, journals and notes are similar to friends sitting in a coffee shop and having a personal conversation. All journal messages and notes are posted on a user’s personal profile page instead of on a community page. There is no single aggregate page listing multiple journal authors or multiple note recipients. Instead, users must go through the personal profile page of an individual user to post a message. The user (or owner) of the personal profile page can control access to journals and notes to “Everyone,” “Only my friends,” or “Only me.” In this personal space, any passerby would be near enough to overhear comments but would immediately interrupt the conversation. The distinction between the sense of communal space and personal space derives from certain architectural elements, which determine the openness to new messages (see Table 2). In a way, these computer-mediated communication formats are similar to MySpace’s and Facebook’s privacy features that allow a user to put a fence around personal property. A unique feature in these health social network sites allows users to add optional health data such as daily weight to forum and journal posts to share with others. Reading new messages also varies across each format; new forum messages are listed collectively on the support community page, and journal and notes are listed on each user’s profile page.

**Table 2:** Comparison of architectural elements in computer-mediated communication formats in MedHelp.

Format	Architectural element				
	Authoring new posts	Commenting	Access and notifications	Privacy settings	Other features
Forum	Any user	Any user	Public Forum Page	Public	Add Tags, Select topic, Add to watch list, Show Ticker
Journal	Owner of journal	Friends (depending on settings)	Profile Page	Everyone, Only my friends, Only Me	Add Tags, Show Ticker, Add Photo
Notes	Friends	None	Profile Page	Everyone, Only my friends, Only Me	Add as friend

By exploring the range of nurturing behaviors displayed across multiple computer-mediated communication formats of an online health supportive community, we can understand how the software features affect communication between individuals of the community. Previous research did not address the communication platform characteristics as an influence on resulting user behaviors. We used content analysis to identify themes in user-created content. We speculated that patterns of nurturant support exchanged across different social media communication formats may vary depending on the architectural elements characterizing the communication tools. In this study we tried to answer the following question: What are the differences in nurturant support types (esteem, network, and emotional) across different communication formats (forum, journal, and notes)? Results from this study would be useful for improving the design of technologies supporting online communities, because the increasing socialization of online health information will open up opportunities for future online health services [4]. By answering these research questions, we hope to better understand the link between site design and group interactions in an online health community.

## Methods

We collected data from a health social media website called MedHelp [44], which has features similar to those in Facebook to help people connect with each other. It is one of the oldest patient communities on the Internet, founded in 1994 by a software developer and pharmaceutical/biotech professional, both of whom were touched personally by their families’ health conditions. Their website has over 12 million visits each month. Alcoholism is one of the leading health problems in the United States, with many researchers publishing studies of the disorder. The Alcoholism Community is one of the biggest communities on MedHelp, with many user activities, which provides enough data for analysis.

Our approach consisted of data collection with a Web crawler and manual content analysis. Although the typology of social support used in this study was originally developed for short conversations between spouses rather than a support group, it has been shown to be sufficiently generalizable for use in support groups [28,33,34,38]. All five of the supracategories from Cutrona and Suhr’s [38] typology were coded in our data. Of these five, we did not find instrumental support, such as the tangible assistance of offering the recipient a loan and offering to perform an indirect task.

### Study Setting: MedHelp Support Communities

MedHelp is a health-oriented social networking service where individuals can connect with other people (patients, caregivers, doctors, etc) and information resources. Its platform provides an environment for registered users to join peer support communities as members and to communicate using several tools, such as discussion forums, journals, and notes. The forum for each peer support community is unmoderated, in the sense that any MedHelp user is able to post questions to the community or respond with comments without undergoing an approval process. The journals format allows users to record thoughts and feelings. Notes are a way for users to keep in touch with each other through their profile pages on MedHelp.

**Figure 1:**
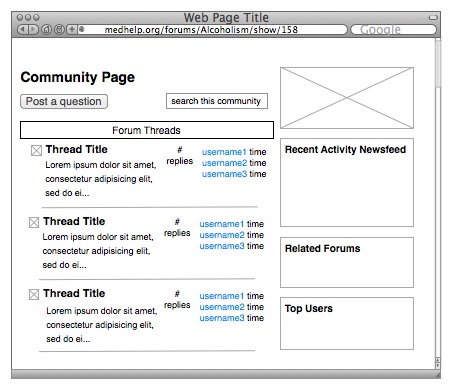
Community activity (messages and updates) are displayed on public forum page.

**Figure 2:**
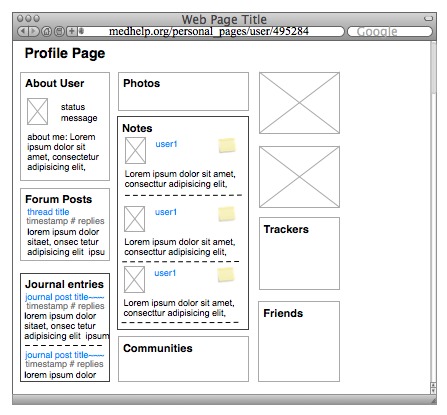
Friends and messages displayed on user’s profile page.

The support community page lists the most recently updated forum threads in the main section and recent activity on the sidebar, which also lists recent updates or journal comments (Figure 1). There is also a sidebar box that lists community members and links to their profile pages. Each personal profile page displays sections of the user’s activity across communication tools (Figure 2). Privacy settings on an individual user’s profile page can affect who can read updates and write notes. If the settings for journals and notes are set to Only my friends, then only users who are friended may view these contents. If the setting is set to Only me, only the user can see his or her own content when logged in. The content in each of these tools is organized chronologically.

#### How Users Message Each Other in MedHelp

**Figure 3:**
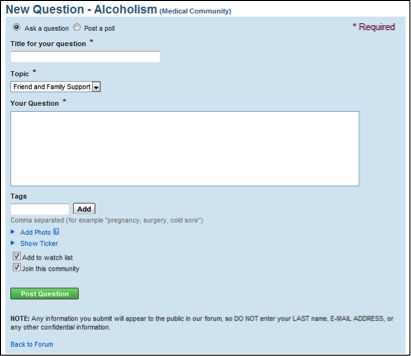
Posting a question on the forum.

**Figure 4:**
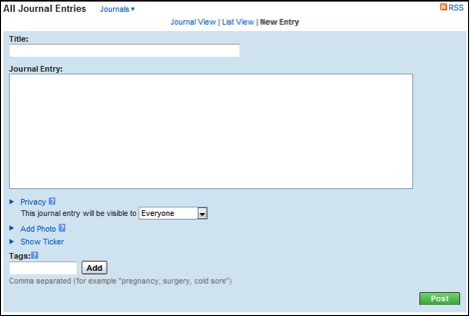
Posting in the journal.

**Figure 5:**
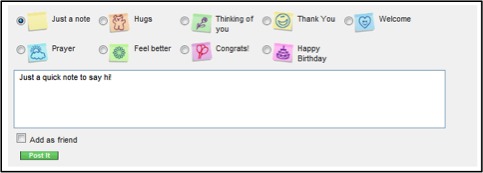
Posting in notes.

The three communication tools we investigated in this study (forum, journals, and notes) are available for any MedHelp support community member to post content. Each tool varies in features for posting content (Figure 3, Figure 4, Figure 5). Users can post questions or polls to the forum, record thoughts in journals, and contact other users with notes.

To post a question in the forum, users are required to type in a title, select a topic, describe their question, and optionally add tags (Figure 3). When posting to the forum it is fairly clear that the intention is for the user to communicate with the entire support community.

A posting to a journal can include title, entry, tags, and photos, with selected privacy options (Figure 4), and is listed only on a user’s profile page. The intention of posting to the journal for MedHelp users is to record their thoughts and feelings. Only the owner of the personal profile page can intiate a thread by making a journal post, but other authorized users are able to comment on a journal post.

Notes on a user’s personal profile page include type of note and the content in the note (Figure 5). Any member who is allowed to read the notes entries is allowed to post. Users can leave notes on other members’ pages if they are allowed to read it. If the user is not a friend, there is an option to befriend the user. However, the owner of the personal profile page is not allowed to post to his or her own notes section.

The journal and notes features are similar to Facebook and MySpace wall posts, which allow users to communicate one-on-one. However, MedHelp is more specific on who can initiate, comment on, and post in journal and notes sections. The screen shots are shown in Figures 3–5.

### Data Collection and Preparation

We developed a Web crawler in the Java language to scrape data from the MedHelp Alcoholism Community [44] and saved the data to text files. The text files were then converted to Excel spreadsheets (Microsoft Corporation, Redmond, WA, USA) for identifying themes during the descriptive content analysis.

The data we were interested in collecting included messages created by members in the Alcoholism Community. We collected publicly available messages from the discussion forums, user journals, and notes on users’ profile pages. Because the forum was slightly different from journals and notes, we took different steps for each sample. To collect forum messages, we had the crawler scrape each thread of messages and divided it into posts and comments. To collect user journal messages and profile posts, we first identified the list of users who publicly listed their memberships in the MedHelp Alcoholism Community. Then on each user’s profile page, we specified that the crawler go to the link for journals to scrape publicly available entries and their corresponding comments. We also specified that the crawler scrape the profile posts. For each message the crawler identified, we attached information, if it was available, about the author, recipient, timestamp, and titles.

### Content Analysis

We used descriptive content analysis to find patterns of social support in messages in the MedHelp Alcoholism Community interactions across 3 months of data (June 19 to September 19, 2009). More than one theme could appear per message. No identifiable information, such as each participant’s drinking problems, was part of the final analysis.

Our unit of analysis was at the message level. We developed definitions of social support types by reviewing examples from the related literature and matched them with themes within the data [21,38,45,46]. A pretest was conducted to determine whether the categories captured a majority of the message postings. Results of the pretest informed our decision to organize our definitions into three categories (information, nurturant, and instrumental). The interrater reliability of the coding system was determined using two coders who were both graduate students in information science with health informatics experience. They received the coding instructions and conducted the coding independently. The results were recorded onto an Excel spreadsheet. We used Cohen kappa to determine the interrater agreement (kappa = .719). This indicated that a high agreement was achieved.

We found only two types of support in the data: informational support and nurturant support. The third type, instrumental support, is typically found in face-to-face support interactions and was not found in our data.

### Support Types: Informational Versus Nurturant Support

Social support is the provision of psychological and tangible resources intended to benefit an individual’s ability to cope with stress, such as information or statements leading the person to believe he or she is cared for. We discuss the types of social support messages used in computer-mediated support groups, organized within Cutrona and Suhr’s [38] guideline for categories of informational support and nurturant support. There are two main types of support: (1) *action facilitation*, which is intended to help stressed individuals to solve or eliminate problems causing distress, and (2) *nurturant support*, which caters to the emotional side by comforting support seekers [36,38]. Action facilitation support includes both informational support and instrumental support. Informational support could be facts, advice, information referral, personal stories, or opinion. Instrumental supports are tangible services, either direct or indirect, for improving the situation (eg, driving a friend to the hospital). In this study we focused on the second type of support occurring in different computer-mediated communication formats of an online health support community because we wanted to better understand how a specific support relates to computer-mediated communication formats and because we found more messages with nurturant support. We looked for patterns of nurturing interactions across each format to identify differences in behavior across the group. Nurturant support includes esteem support, network support, and emotional support, which are defined in more detail in the next section.

### Coding Scheme: Informational Support Types

Informational support in messages conveys instructions, including (1) advice or teaching, (2) referrals to other sources of information, (3) situation appraisal, (4) stories of personal experience, and (5) opinions. Messages coded as informational support often appeared as an attempt to reduce uncertainty for the message recipient [28,34,38]. We identified these informational support types in the forums, journals, and notes.

### Coding Scheme: Nurturant Support Types

Nurturant support posts provide expressions of caring or concern [30,33,34,38,47,48]. These are summarized in Table 3. Nurturant support is a more compassionate type of support whose purpose is to help the recipient with coping or relieving stress. It has been studied in a variety of online patient support communities, listed in Table 4 [6,19,21,28,31-34,37,49].

**Table 3:** Definitions of nurturant support types for content analysis measures.

Support type		Definition	Example
**Esteem**		Gives positive comments validating a recipient’s self-concept, alleviating feelings of guilt as a person; includes compliments, validation, and relief from blame [36]	*Congratulations on your sobriety!*
	Compliment	Conveys positive assessment toward someone or emphasizes the recipient’s skills and abilities	*Thanks x. This is a great journal entry. Thanks for the laughs. I really needed it. My favorite one is #16.*
			*Good Job! I’m sooooo proud of you =0) :o) =)*
	Validation	Recognizes need by expressing agreement with the recipient’s perspective on the situation	*X, no need to apologize its a great post especially when it comes from your heart <3*
	Relief of blame	Tries to alleviate the recipient’s feelings of guilt about the situation	*Its not your fault*
			*Don’t blame yourself*
**Network**		Focuses on messages to broaden support seekers’ social network so they don’t feel alone, by connecting them to others with similar situations; includes access, presence, and companions [36]	
	Access	Invites new members to join conversations or offered to connect members with others having similar interests	*Well, I guess I wasn’t much help, but I appreciate the input, and it’s good to know you’re not alone. Thank you x. Maybe we can help each other.*
	Presence	Offers to spend time with the person, to be there in time of need	*...well my dear...please stay in touch with us here...we do care what happens to u!:)*
	Companions	Reminds the person of availability of companions, of others who are similar in interests or experience	*Just reach out and I will be there ok?*
**Emotional**		Gives expressions that support recipient’s feelings or reciprocates emotion; the emphasis of this category is on supporting emotional states rather than the recipient’s identity or self-concept [36,46]	*You’re going through a rough time*
			*Hang in there hon.*
	Relationship	Stresses the importance of closeness and love in relationships the recipient has with others	*I have missed this forum SO much! Finally back from crazy land and look forward to seeing all those familiar names comment on the questions.*
	Physical affection	Offers physical contact, including hugs, kisses, hand-holding, shoulder patting; obviously, physical affection could not be given online, but it was often offered and conveyed verbally	*You deserve a big bear hug!*
	Confidentiality	Promises to keep the recipient’s problem in confidence; confidentiality is typically symbolic [28]	
	Sympathy	Expresses sorrow or regret	*Sorry it had to happen to you.*
	Listening	Provides attentive comments as the recipient speaks	
	Understanding and empathy	Expresses understanding of the situation or discloses a personal situation that communicates similarity of one person’s experiences with another’s	
	Encouragement	Provides messages of hope or confidence	*Hang in there!*
	Prayers	In spiritual words, mentions praying, spiritual healing, or God	*I will keep you in my prayers*.

**Table 4:** Comparison of nurturant support types in previous studies (by reference number).

Support type		Studies and settings						
		Disability	Eating disorder	HIV/AIDS^a^	Addiction	Torn knee ligament	Depression	Cancer
**Esteem**		[28]	[6,33]	[32]	[31]			[49]
	Compliment	[28]		[32]	[37]			
	Validation	[28]		[32]			[34]	[21]^b^
	Relief of blame	[28]		[32]				
**Network**		[28]	[6,33]	[32]	[31]		[34]	
	Access	[28]		[32]				
	Presence	[28]		[32]				
	Companions	[28]		[32]				[21]
**Emotional**		[28]	[6,33]	[32]			[34]	[49]
	Relationship	[28]		[32]				
	Physical affection	[28]		[32]				
	Confidentiality	[28]		[32]				
	Sympathy	[28]		[32]				[21]
	Listening	[28]		[32]				
	Understanding and empathy	[28]		[32]	[31,37]	[19]		[21,49]
	Encouragement	[28]		[32]		[19]	[34]	[21,49]
	Prayers	[28]	[6]	[32]			[34]	[49]

^a ^Human immunodeficiency virus/acquired immunodeficiency syndrome.

^b ^This study used the label emotional support for validation support.

## Results

The three samples of data consist of user-created messages from the discussion forums (n = 493), user journals (n = 423), and notes (n = 1180). Based on the displayed structure, forum and journal messages were grouped into *posts *(ie, messages that start the thread) and *comments *to the post. The data sets encompassed 81 forum posts; 412 forum comments, 88 journal posts, and 335 journal comments (Table 5).

**Table 5:** Summary of samples and their sizes.

Type of message	Sample		
	Forums	Journals	Notes
Messages	493	423	1180
Posts	81	88	NA^a^
Comments	412	335	NA

^a ^Not applicable.

There was a range of message characteristics. A message contained on average 2.57 codes with a maximum of 10 codes per message, except for the first post of each thread, which had a maximum of 6 codes. Some messages only offered support (eg, “Have you tried Naltrexone? It is sup[p]osed to help with the cravings there are other meds that can help with it too. If all else fails, make a picture of tea and pop some popcorn and hang out with him with your ‘drink’.”), or only requested support (eg, “Hi, is there a medicine to take to stop the craving for alcoholic drink?”). We first identified informational and nurturant support in the samples (both provided and requested) and present these findings first before presenting specific nurturant support types.

### Informational Versus Nurturant Support

The contrast between informational and nurturant support was apparent among the computer-mediated communication formats. Discussion forums were more informational than the other two (journals and notes). More nurturant support was offered in notes and journal comments, whereas more informational support was offered in the forum. However, when requesting social support, users were not as likely to seek nurturant support. The only exception is in journal posts, where the user was more likely to seek nurturant support.

**Figure 6:**
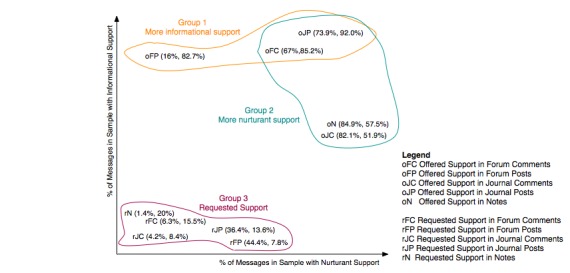
Informational and nurturant support levels in user-created messages.

To illustrate the differences in the computer-mediated communication formats, we plotted data onto a graph (Figure 6). The points are noted in parenthesis (x,y), where x indicates the number of messages in the sample with nurturant support and y indicates the number of messages in the sample with informational support. The sample name has a prefix that signifies offered support (o) or requested support (r). Three main clusters showed up. *Group 1 *contained formats where a majority of messages offered informational support (eg, advice). This type of support was found in 83% (67 of 81 messages) of forum posts, 85.2% (351 of 412 messages) of forum comments, and 92% (81 of 88 messages) of journal posts. *Group 2 *contained formats where a majority of messages offered nurturant support (eg, encouragement). This type of support was found in 74% (65 of 88 messages) of journal posts, 67.0% (276 of 412 messages) of forum comments, 84.9% (1002 of 1180 messages) of notes, and 82.1% (275 of 335 messages) of journal comments. *Group 3 *contained all the formats’ levels of requested information and nurturant supports, which were relatively low compared with offered supports.

Our initial analysis showed more messages with nurturant than informational support. This is an interesting type of support to investigate because it tends to be more interactive in a bidirectional way. We were interested in studying this to have a better understanding of how a specific social support can be facilitated. In our next step, we analyzed the specific types of nurturant support more carefully for each sample, and then compared results across all samples. For example, there was a link between requested support in journal posts (37%, or 32 of 88 messages with nurturant) and offered support in journal comments (82.0%, or 338 of 412 messages with nurturant). There was an interesting connection in reverse for notes, where users requested information but provided more emotional support. While forums may be seen as a question-and-answer portal for exchanging information, the portion of nurturant support was higher than expected in the comments.

### Nurturant Support Offered

**Figure 7:**
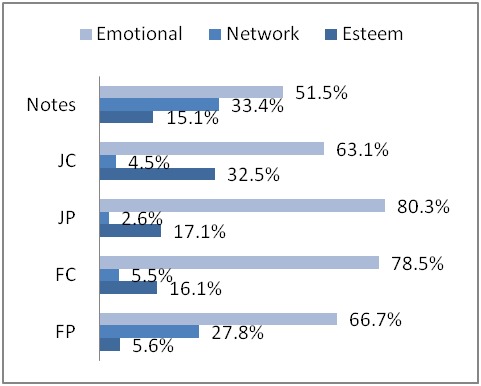
Nurturant support offered in messages. FC = forum comments; FP = forum posts; JC = journal comments; JP = journal posts.

Table 6 summarizes the number of messages showing nurturant support offered for each sample. After tabulating these numbers, we converted them into percentages. Figure 7 displays the percentages and shows two types of patterns. Two nurturant support patterns emerged in messages that offered support: emotional > network > esteem (forum posts, notes) and emotional > esteem > network (forum comments, journal posts, journal comments). We explain these two patterns in the section.

**Table 6:** Number of messages offering nurturant support in each format.

Support type	Forums		Journals		Notes
	Posts	Comments	Posts	Comments
Esteem	1	53	13	124	220
Network	5	18	2	17	488
Emotional	12	259	61	241	752
Total	18	330	76	382	1460

Emotional was the most commonly appearing subtype among offered nurturant support (Figure 7). Network and esteem occurred less in comparison. In two sets (journal comments, forum comments), esteem is greater than network (esteem > network). This pattern may reflect the compassionate nature of users who recognized the perspective of the first author, compliments, or relieving blame. In addition, journal posts also displayed more esteem than network, which may indicate their authors’ awareness of their audience. Conversely, in the samples that had more network than esteem (notes, forum posts), the strategy might have been to increase communication with an emphasis on presence, access, or companionship.

The first pattern where network is greater than esteem support might be an indication of the user’s informational or emotional state while starting a thread in the forum or creating a note for a friend. The emphasis on network over esteem suggests promoting presence and involvement with the community, which could be a strategic expression for being a worthy recipient of social support. The lower amount of esteem support might suggest social status as less important than membership. The similarity between forum posts and notes may suggest that users post without expectation of a direct response.

The second pattern, where the communication formats had the pattern of more esteem than network support (emotional > esteem > network; forum comments, journal posts, journal comments), may suggest that these formats are more suitable for praising and complimenting others. Forum comments contained less explicit network support, which might suggest that the act of replying shows presence. Similar to journal posts and journal comments, here the act of posting may be an indicator of network support. Offering emotional and esteem support more than network might result from an assumption that other members are aware of network support, and need is not explicitly stated. It is possible that users were compelled to offer esteem support when they were more familiar with someone or his or her situation.

One possible cause of pattern differences could be that levels of network or esteem support are correlated with relationship strength. Surprisingly, offered support in journals is different from that in notes, even though their features make them “publicly private.” While journals and notes users who communicate with each other might be friends of each other, the longer message format of journals may not be as conducive as notes to maintaining relationships.

#### Nurturant Support Requested

We identified fewer messages with requested nurturant support than offered nurturant support (Table 7). We calculated the percentage by dividing the number of messages containing nurturant support by the total number of messages.

**Table 7:** Number of messages requesting nurturant support. in each format

Support type	Forums		Journals		Notes
	Posts	Comments	Posts	Comments
Esteem	6	8	21	8	2
Network	8	10	0	0	6
Emotional	29	10	23	7	9
Total	43	28	44	15	17

For requested nurturant support, emotional support was highest in all sets. Esteem support was also frequently requested among all sets of messages but was most noticeable in journal comments, which may indicate a desire on the part of the commenter to help the journal message author feel better about him- or herself. Journal messages did not show network support, possibly because readers were already known friends. In comparison with other formats, in notes and forums requests for network support are quite frequent.

The three patterns that emerged among messages that requested nurturant support were emotional > network > esteem (forum posts, notes); emotional = network > esteem (forum comments); and emotional = esteem (journal posts, journal comments).

The first of these patterns (emotional > network > esteem) appeared in forum posts and notes. The combination of requested supports was an effect of users explicitly stating the type of support they sought, such as an emotional release from thinking about the situation. Notes had more messages requesting network than forum posts, which might emphasize referring to the friendship between the author and receiver. In the forums, more comment messages offered network support than in post messages, which could indicate that people comment on forum threads because they know there is someone else with a similar situation to talk about. The pattern of requested support in notes is most similar to that in forum posts, where network was requested more often than esteem, possibly because they were emphasizing their presence in the online community.

In the second pattern, emotional support occurred in the same number of messages as esteem (forum comments), a demonstration of users showing empathy and appreciation. Perhaps members found talking to each other soothing, especially in the encouragements and time spent chatting with each other. While there was less emotion in forum comments than in forum posts, more esteem and network supports were requested. Perhaps it was easier for members to ask for additional types of support after asking for emotional support at least once before.

We observed the third pattern of requested nurturant support in journal posts and journal comments, where users were more likely to request only emotional and esteem, rather than network. Because users who write to each other in the journals have a higher likelihood of being friends in the online community or have a stronger relationship than forum users, they may find it unnecessary to emphasize reminders of network presence, as that might be a purpose more suited for notes. In the case of no network support in a pattern, the architectural features of the tool offered a way out of explicitly stating network support in the note content through the friends feature shown on the profile page. In the journals, users intend to write for themselves or friends, and usually only friends notice new posts and are willing to comment after reading them.

## Discussion

**Figure 8:**
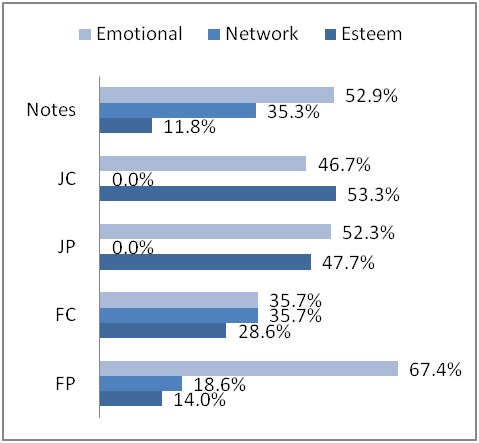
Requests for nurturant support in messages. FC = forum comments; FP = forum posts; JC = journal comments; JP = journal posts.

This study identified nurturing social support types in user-created messages across three different text-based communication tools (discussion forums, personal journals, and notes) of an online health care social networking site. The content analysis codes came from a literature review and were organized into Cutrona and Suhr’s [38] supracategories, and are similar to those from other studies [33,34]. We analyzed the social support in the MedHelp Alcoholism Community most often exchanged by group members during a 3-month period. Interestingly, the nurturant support pattern in forum posts and notes was the same for both the support offered dimension and the support requested dimension, where emotional was greater than both network and esteem support (emotional > network > esteem). These percentages are shown in Figure 7 and Figure 8, and a summary of patterns is explained in Multimedia Appendix 1. The patterns (emotional > esteem > network; emotional = esteem) we found among journal messages (journal posts, journal comments) also group these samples together. Forum comments is the only sample where the pattern of offered support (emotional > esteem > network) was different from requested support (emotional = network > esteem). While the three computer-mediated communication formats of MedHelp have similarities, the differences in architecture appear to have affected the support exchanged.

We found that some formats were more conducive to emotional connecting than others, yet overall each was used for different purposes. This study gathered empirical data of patterns varying nurturing support among the different online tools. This information could be potentially useful to social support scholars and designers of online support groups to understand how software features affect users’ behavior. In this section, we explain the results in the context of theories from the related research literature.

### Comparing Patterns From This Study With Related Studies

Similar studies identifying social support in online health support communities found various levels for each nurturant support type [6,19,21,28,32-35]. These studies collected data from settings using bulletin boards or email lists, which is similar to the MedHelp forums. To the best of our knowledge, there have not been any documented cases of health support groups incorporating computer-mediated communication formats similar to MedHelp’s journal or note formats, nor any studies of social support within them. Many studies reporting social network site data are not tailored to a health issue, but rather are general social network sites (eg, Facebook, MySpace, and LinkedIn), where users may already know each other offline. Table 8 summarizes results from other studies identifying social support in health communities in bulletin board or email list styles, rather than social media features such as profile post messages and journal entries. Most studies found that emotional and network support appeared more frequently than esteem support.

**Table 8:** Nurturant information support interaction patterns in related studies.

Study	Data	Architectural element	Pattern
McCormack [6]	Anorexia bulletin board	Bulletin board	Emotional > network > esteem
Preece [19]	Torn knee ligament, 500 messages, April 1996–April 1997	Email list	Nurturant > information (no distinctions)
Bambina [21]	Support OnLine Cancer Forum, 84 members,1149 messages (unmoderated)	“an Internet cancer support forum...a virtual space...[to exchange] support;” only requires email address; archives posted online and publicly available	Network > emotional
Braithwaite et al [28]	Support network, 42 users, 1472 messages	“Messages were distributed via email through a nationwide computer BBS network.”	Emotional > esteem > network
Meier et al [31]	10 cancer mailing lists hosted by the Association of Cancer Online Resources (5 months)	Mailing lists	Nurturant > information (no distinctions)
Cunningham et al [32]	Alcoholism, 10 months, 474 posts (moderated)	Bulletin Board	Emotional > esteem
Coursaris and Liu [33]	HIV/AIDS^a^, 5000 messages	Bulletin Board	Emotional > network > esteem
Eichhorn [34]	5 eating disorder message boards, 490 messages	Yahoo Discussion Groups	Emotional > network > esteem
Pfeil and Zaphiris [35]	Depression/seniors	Bulletin Board	Network > emotional > esteem
Selby et al [36]	Smoking cessation	Web assisted tobacco intervention, bulletin board	Esteem > emotional

^a ^Human immunodeficiency virus/acquired immunodeficiency syndrome.

The members of the Alcoholism Community favored the emotional type of nurturant support, similarly to findings from other health support communities [6,19,28,33,34]. We also noted a substantial amount of informational support in our data in addition to nurturant support. Of all our results, the forum posts sample appears to be most similar to previous findings from bulletin boards (emotional > network > esteem). Notes showed a similar pattern to the forum posts; however, journal messages followed a different pattern with no network support (esteem = emotional). This low amount of network support shows that it is not typical to use journals as a place to indicate a relationship bond between two users, or that network support needs were met simply by participating in the community without the need for explicitly stating requests or offers of network support. In notes, on the other hand, any member can leave a note for a friend and not expect any response. With the journal format, users are given more control over who can read and comment, so in that format initiating an explicit support request is required to receive any comments. Journal post authors are more likely to post a message for themselves. For example, one author wrote in her journal “i doubt if anyone will read this and that is ok as I am writing this more for my benefit then anyone else...” and probably did not expect a reply. In fact, the same post goes on to state “I do not need anyone to tell me how lucky I am, how well off i am, or how good i look. I still feel very fragile and needed to just let someone know that might have a word or encouragement or knidness [sic]...”

### Explaining These Patterns

Although offline support is often available from friends and family, e-patients and their caregivers participate in online support groups for the added benefit of specific types of nurturant support. We found different types of nurturing support in the MedHelp Alcoholism Community of varying patterns across the multiple computer-mediated communication formats. Two theories might be able to explain why the number of support types varies: (1) the purpose of communication affects which format people use to convey and seek help for different types of needs, and (2) the public and private spheres where communication is mediated by particular norms of acceptable behavior affect the content construction for each message.

### Limitations

Our results cannot be generalized for two reasons. First, the study setting here is more narrowly defined than in previous studies and we were studying a specific alcoholism community from MedHelp. Second, MedHelp has particular software features for computer-mediated communication that other websites may not have. Alcoholism as a health condition has its own characteristics that can influence attitudes and behaviors. Our hope is to extend more work on each of these reasons to better understand the impact of technology on human interactions.

### Purpose of Communication

People use different communication tools for different purposes; for example, some online community members sought information, while others sought compassion and intimacy through conversations [22,50]. This distinction is possibly the result of numerous waves of information needs when recovering from alcoholism [7]. Presenting one’s information need(s) to the community may be a way to initiate presence and involvement as a new member, but also for older members to welcome new members. While information is often explicitly stated within messages, sometimes participation is motivated by other reasons such as relationship maintenance [10,23]. In fact, results from our analysis of notes messages support previous findings from MySpace profile wall posts, which mostly contained short messages to fulfill two purposes: making initial contact and keeping in touch [23]. Because it is so easy to publish information with social media technology, blogs are often used as way to share knowledge and skills, rather than to keep in touch with friends and family [51].

While the purpose of communication can vary across computer-mediated communication formats, it is not the same as the purpose of the community—that is, exchanging support. In this case, the purpose of communication through notes and forum posts (eg, reaching out to others with emotional and network supports) and the purpose of the community (eg, connecting with other patients) overlap. For example, access to other patients’ stories on the Internet can be reassuring [52]. In addition, social media make it easier to obtain social feedback and reviews. People who are unsure about medical answers find confidence from social feedback [37]. Health issues trigger anxiety and questions; however, online communication with familiar folk can be soothing, as it might enhance the quality of relationships and improve the psychological well-being of the support seeker [29,53]. For example, most blogs allow readers to leave comments and, in this way, they both generate conversation and encourage collaboration [51]. Users of online support communities often communicate in one-on-one situations or in small groups of 3–5 individuals [29].

### Communal Versus Personal Spaces

The different nurturant support patterns in the various computer-mediated communication formats may also be explained by communication theory, which separates communication into that targeted to the public (eg, mass media, advertisements) and private interpersonal communication (eg, email). In recent years, studies of online communication have shown that social media mimic physical spaces by providing online spaces for communication but are also used to exchange private information. The distinction between the traditionally public and private spheres is blurred in online communication [9,17]. We believe that, because privacy can be controlled through notes and journals, we can distinguish these as private spheres, which are more personal, and the forum as a public sphere, where conversations are exchanged in a community setting. While the sphere may be a factor that influences behavior in each format, we believe that the computer-mediated communication format itself is the driving force for different behavior patterns. For example, notes are similar to forum posts, where as forum comments are similar to journal posts and journal comments. It is possible that people are not aware of privacy issues in an online environment or do not find it necessary to control privacy settings or learn about their implications.

In a physical setting, it is easy to perceive the relative privacy of the space. However, in an online environment, the amount of privacy is not transparent, and many private areas become “publicly private.” In this case, perhaps the MedHelp users did not assess the online setting as they would a physical face-to-face setting. In light of the content observed through this community (eg, blackouts, possible violent episodes), the online setting diminishes the amount of stigma that would be present in face-to-face support.

### Emerging Issues: Collaboration and Health Outcomes

In the course of identifying support types, we also found evidence of users in the Alcoholism Community mentioning their health outcomes (eg, quitting drinking) or of collaboration. Although bulletin boards have a less-than-expected effect on behavior change such as smoking cessation, they are often an integral component in Web intervention programs by allowing participants to communicate with each other [54]. Bulletin boards are not suggested as a primary information source or to be appropriate for everybody, as only certain people will voluntarily use them, and they often have a small core set of active users. Participation is also linked to other factors such as an e-patient’s phase of quitting (ie, former smokers will have higher participation rate than those prequitting) and the speed of responses to posts.

Social media allow a direct connection between information and the consumer [55]. Social networking enables and facilitates collaboration and collaborative filtering processes. For example, it enables users to see what their peers or others with a predefined relationship (friends, colleagues, fellow patients, etc) are doing; enables automated selection of relevant information (based on what peers are doing and reading on the Web); enables reputation and trust management, accountability, and quality control; and fosters viral dissemination of information and applications (it is this viral marketing aspect that makes Web 2.0 applications so attractive to venture capitalists and public health practitioners alike). Moreover, social networking is a potentially powerful tool to engage users, in that it provides social incentives to enter, update, and manage personal information. Teenagers spend hours keeping their Facebook profile current, constantly updating their status. The same generation of users may possibly turn their attention and energy to similar tools for health.

### Future Work

More research to better understand how social support is communicated among computer-mediated communication formats in these groups would be particularly useful to those interested in designing, providing, using, or evaluating online support as an alternative to face-to-face support. Knowing how to develop and sustain an online community is important, as a certain level of active users from peer families and visible signs of their activity are necessary to attract returning users [56]. Potential benefits of social network site participation for e-patients include peer support (availability of an opportunity to receive and offer support), which can be an empowering experience in a customizable setting (eg, revealing limited identity information or restricting the number of friends). Not only that, a personalized space could also help patients open up about their health issues. This could be beneficial to the area of preventive medicine.

Future work that identifies patterns at the message level rather than patterns for each sample would help with understanding the construction of each message. This study did not account for individual characteristics such as each member’s support profile, such as whether he or she is more likely to provide or request support. Future work that identifies whether support is given explicitly to a recipient could be useful.

Another direction for future work is to explore theories on personas at an individual level. For example, the theory of faceted social identity in sociology states that people behave differently with different groups. Users communicate with different categories of relationships online [57]. Users present identities depending on who they are communicating with and seek different types of support. For example, close friends are more likely sources of emotional support. Email is thought of as more personal and private than social network sites, and users are more likely to experience more comfort in using that platform to communicate with close relations [57]. Some users may also be motivated to keep in touch with others, whereas others want to share information [22,50,51]. Age can also be a factor in activeness online.

Yet another research angle for studying patient communities is shared patient data, which is mostly quantitative data (eg, blood pressure) rather than qualitative data such as stories and advice [49,58]. Participants often look for similar patients (by having a medical ailment, hobby, or other thoughts in common) to make their connections. Websites such as PatientsLikeMe “promote data-centered patient conversations.” This might be a good direction, as there are many lurkers in support communities [59], who feel that reading community messages is enough to feel a sense of belonging to the collective intelligence. The influence of interpersonal association on personal health records could lead to improved health outcomes as people become more aware of their day-to-day behaviors.

### Conclusion

People are drawn to online health support communities because of the availability of tailored information and the opportunities to meet peers who share similar experience(s). Emotional support is an important component of interactions within support groups; however, it varies in exchange across different social media communication formats for reasons such as demographics and formats of communication tools. Users may prefer to ask basic informational questions in the forum because they would rather have any answer than only the specific answer. Furthermore, users may prefer using the journals to disclose specific information that only a select few people can read. Deeper understanding of these associations would help in designing health-related Internet applications. This in turn can contribute to applications such as online intervention programs.

We found two general patterns in offered nurturant support and three general patterns in requested nurturant support. Offered nurturant support is typically emotional support such as encouragement, and then either esteem (eg, validation) or network (eg, reminders of presence) support. Requested support was also typically emotional support, but with more network than esteem support. There was no mention of network support in the journal posts or comments. We attempted to link theories with results to explain the supportive behaviors. Theoretical perspectives include (1) the purpose of communication, where people use different platforms differently to convey different types of information, and (2) the influence of public and private spheres of communication on the users’ behavior. Further research could provide more insight into these two phenomena.

This research offers a novel message regarding the impact computer-mediated communication format has on user interaction patterns in online support communities. It is not clear how people seek or provide social support in an online format, so we explored how the social media platform facilitates the exchange of social support. Social media have more computer-mediated communication features than were present in previously studied software platforms for online communities, in that they give the user a bit more control over whom they share information with, by offering multiple formats for private and public messaging. This research also examined the issue of space preference for privacy and the kinds of support in each format for disclosing information to specific people. We studied how people use social media for nurturant support to have a better understanding of how computer-mediated communication formats can encourage a specific type of social support. For example, people with alcoholism, people who want to quit smoking, or cancer survivors may need more nurturant support. People using other types of health intervention such as weight loss may need more informational support. Therefore, the design and utility of social media can be tailored to the particular purpose.

## Multimedia Appendix 1

Summary of offered and requested interaction patterns.

## Abbreviations

HIV/AIDS: human immunodeficiency virus/acquired immunodeficiency syndrome
