# Supplementary material for: Interaction Patterns of Nurturant Support Exchanged in Online Health Social Networking
Source: J Med Internet Res. 2012 May 3;14(3):e54. doi: 10.2196/jmir.1824 (PMC3799482; doi:10.2196/jmir.1824)
Supplement: Supplementary file 1 [file jmir_v14i3e54_app1.pdf]

Patterns	Offered	Requested
EM>EST>NET	Forum Comments, Journal Posts, Journal Comments	
EM&EST		Journal Posts, Journal Comments
EM>NET>EST	Forum Posts, Notes	Forum Posts, Notes
EM=NET>EST		Forum Comments

Table seq . Summary of offered and requested interaction patterns
